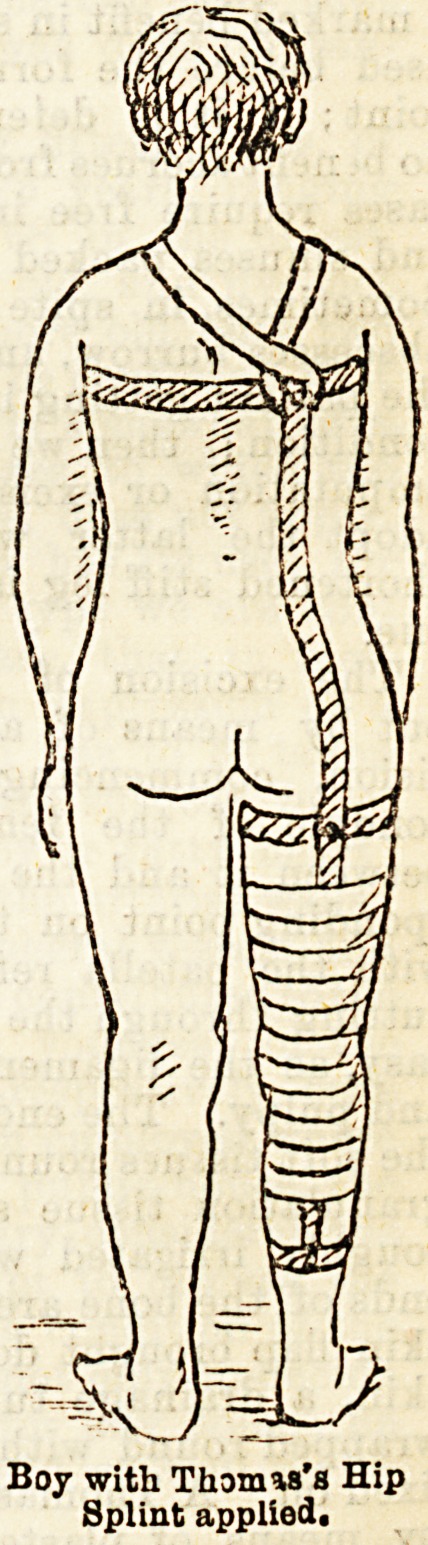# Treatment of Tubercular Joints

**Published:** 1893-05-06

**Authors:** 


### THE HOSPITAL CLINIC.

[*The Editor will be glad to receive offers of co-operation and contributions from members of the profession All letters should be addressed to* THE EDITOR, THE LODGE, PORCHESTER SQUARE, LONDON, W.]

### ROYAL SOUTHERN HOSPITAL, LIVERPOOL.

### TREATMENT OF TUBERCULAR JOINTS.

In describing the treatment of tubercular joints we shall confine ourselves to the larger joints, as it is these joints that the disease most frequently attacks. Take, for example, the hip joint, how common is disease of this joint compared to any of the other joints. Why this should be the case we have no opinion to offer, nor are we concerned at the present time with this aspect of the question. We must confine ourselves simply to the treatment of these affections, and in doing so we shall deal first of all with the treatment of the joints in their early stages and then in the later stages.

*Hip Disease, or Tubercular Disease of Hip.*—In these cases, where we are fortunate enough to receive the patients in the early stage of the disease, and by the early stage we mean when the very first symptoms present themselves, such as limping, pain in the joint, and tenderness, with, perhaps, fixity of the limb—in these cases, then, absolute rest is the first essential, and in a great number of cases extension is also applied. The little sufferer (for it is generally children who are the afflicted ones) is at once confined to bed, and the extension by weights commenced. This is managed as follows: Two pieces of strapping are taken about twelve or eighteen inches long and a half to two inches wide (according to the size of the limb), and these are applied, one on either side of the leg, commencing just below the tuberosities of the tibia; to the other end of the strapping some webbing is attached, with buckles fixed to it. A piece of wood, three inches by two inches, is then taken; this is to form what is called the stirrup for suspending the weight by, there is a hole bored in the centre, and to each end a piece of webbing is tacked; this is buckled to the webbing at the end of strapping, through the hole in the wood a piece of cord is passed and fixed, to this the weight is attached, the weight varying from two to seven pounds, according to each individual case; the foot of the bed is elevated some four or five inches, so as to enable the weight of the body to act as the counter extension. We prevent the movement of the trunk by means of sand-bags laid over the sheet that covers the patient, one on the outer side of the affected limb, extending from axilla to foot, one on the inner side, from the perinæum to the same point; while on the healthy side of the body another bag is placed, so as to reach from the axilla to the hip, and by these means preventing improper movement both of the trunk and of the diseased limb. Counter irritation to the diseased hip we sometimes employ, such as blisters or tr. iodi painted over the part. We do not allow the children to remain in bed for long in these early cases—at the very longest ten days or a fortnight—and directly they get up they are fitted with a Thomas's hip splint. We are particular about the fit of the splint; it extends from the angles of the scapula to about the middle of the leg, the upright is not too thick, but sufficiently so to prevent any bending, the curve for the buttock is nicely fashioned to prevent any pressure; the cross-pieces are three in number, fixed at right angles to the upright, one for the thorax, one for the thigh, and one for the leg; these cross-pieces are somewhat pliable, in order to allow of adapting them to the parts. The splint when in position should pass down the thigh between the great trochanter and the tuberosity of the ischium. The cross-piece for the thorax has two holes, one in each end, for the passage of a bandage to fix it accurately to the chest, and pieces of webbing or bandage are attached to it and passed over the shoulders to prevent the splint slipping down. The splint having been fixed in the way mentioned, the patient is allowed to get about on crutches with a high-soled boot fitted to the foot of the sound leg, and in this way allowed to leave the hospital, if possible, for the country, and to show himself for inspection at first every month, then at longer periods, until a cure is complete, which generally takes place from eighteen months to two years, and we impress upon the friends the necessity and importance of the prolonged use of the splint, for should this be discontinued too soon, a relapse, perhaps with suppuration, may occur. Should the patient show any constitutional dyscrasia, we generally prescribe ol. morrhuæ or syrup hypophosphite. This is the routine treatment in these very early cases of hip disease. As we before remarked, we unfortunately do not have the opportunity of treating these cases in their early stage, except in a very few. The majority have progressed to the second or third stage, in which we have extreme flexion, with adduction or abduction. The buttock is flattened and wasted, there is apparent lengthening or shortening, starting pains are much more frequent, especially at night. It is in these cases particularly that we see the beneficial effect of extension, and consequently it is the very first thing that is done, applied as before stated; it relieves the most distressing of all symptoms, the night startings. The position of the limb is also greatly improved after some time, which will probably be a month or five weeks. Accordingly, as the night startings subside, the extension is changed for the Thomas's splint, and the patient allowed to get about on crutches.

Boy with Thomas's Hip Splint applied.

We should here remark that some cases are put at once into the Thomas's splint without the weight extension being used at all. If the malposture be strongly marked, an anæsthetic is administered, the limb gently brought into a proper position, and the splint at once applied. Then we come to the more advanced cases which go on to suppuration and the formation of an abscess; here the practice also differs somewhat, in some the abscesses are not interfered with; if they open themselves all well and good. In other cases again, as soon as pus is detected, it is let out by means of a free incision, and any carious bone scraped away and removed, the cavity syringed out with some antiseptic lotion, and a drainage tube inserted and allowed to drain into antiseptic dressings, or instead of a drainage tube, the sinus is packed with iodoform gauze and dressed every second or third day. These, of course, are the advanced cases, and require absolute rest in bed until all signs of active mischief have subsided. It is rare for excision of the hip joint to be carried out at the above hospital. When such treatment is called for it is done in the ordinary way with the usual incisions. This generally is the treatment in the various stages of hip disease. The results, we must admit, are not altogether satisfactory, but we fancy they are as good as by any other method of treatment; in any case, we are very much afraid that only a few survive and reach adult life. If they are not carried off by disease of the hip, tubercular disease of the lungs supervenes and causes death. A method of treatment we have tried just lately is the application of severe counter irritation in the form of Pacquelin's thermo cautery, and this is drawn over the joint in parallel lines, and then again more parallel lines crossing these at right angles until a large surface is involved. It is more particularly in knee and elbow cases that we have tried this method of treatment, and with very favourable results.

Now, with reference to those cases of strumous disease of the knee joint. In these cases the joint is absolutely fixed by one of the numerous splints that are in use for the purpose. We generally use a Thomas's knee splint, and if there is simply induration and thickening of the synovial membrane and fringes without any breaking down in the joint and the formation of pus, we apply very often Pacquelin's thermo cautery, as before mentioned, and dress the wound thus made with ung. boracis, being sure that the limb is fixed before the cautery is applied. This certainly has a marked benefit in some of the cases, but it must be used before the formation of pus in and around the joint; if it is deferred till this happens no benefit accrues from the practice. These cases require free incision and the cavities and sinuses packed with iodoform gauze. Sometimes, in spite of this treatment, the abscesses burrow, and the disease advances, the patient getting into a miserably hectic condition; then we have to fall back on amputation or excision, and we generally adopt the latter with the idea that a shortened stiff leg is better than a wooden one.

The excision of the joint is carried out by means of a horseshoe-shaped incision, commencing from the internal condyle of the femur, round beneath the patella, between it and the tubercle of the tibia to the corresponding point on the external condyle, and the skin with the patella reflected, the joint disarticulated by cutting through the crucial ligaments. This is quite easy, as the ligaments of the joint are generally soft and pulpy. The ends of the bones are separated from the soft tissues round and sawn off, all the soft, pulpy, granulation tissue scraped away, and the joint thoroughly irrigated with 1 in 3,000 perchloride. The ends of the bone are then brought into apposition, the skin flap brought down and stitched to surrounding skin, a drainage tube inserted, and the whole limb wrapped round with antiseptic cloths till the splint is fixed on. A Thomas's knee splint is fixed to the limb by means of plaster of Paris above and below the excised joint to make it firm, and prevent any movement whatsoever. The foot is carefully placed at right angles to the limb, and fixed in this position by means of plaster of Paris also. The antiseptic cloths are now taken off, the foot washed and dressed with double cyanide gauze and salicylic wool, and not disturbed for twenty-four hours, unless the dressing is soaked through with discharge, or pain or rise of temperature calls for a re-dressing, after which time it is dressed every second or third day, or every day if necessary. The patient is kept in bed until the bones are united firmly and all old sinuses have healed. He is then allowed to get up, still wearing a long Thomas's knee splint with a calliper attached, to prevent any weight being borne on the recently united bones. He will, of course, have to use crutches. We can call to mind certainly two cases where the operation has been a very great success, each patient having a stiff leg, of course, but a very sound one, and also being improved in general health, looking fat and well, and delighted with the stiff leg.

We need not emphasise the importance of removing these patients from the hospital atmosphere as soon as possible, for we feel convinced that, were this accomplished earlier, a greater number of cases, not only of strumous joints, but many other diseases would give a more favourable result. How a hospital atmosphere affects the healthy is a well-known fact; how much more will it affect those brought low by disease?

## Figures and Tables

**Figure f1:**